# Reference-free phylogeny from sequencing data

**DOI:** 10.1186/s13040-023-00329-x

**Published:** 2023-03-27

**Authors:** Petr Ryšavý, Filip Železný

**Affiliations:** Department of Computer Science, Faculty of Electrical Engineering, Czech Technical University in Prague, Prague, Czech Republic

**Keywords:** Sequence similarity, Phylogeny, Levenshtein distance, Reads, Contigs

## Abstract

**Motivation:**

Clustering of genetic sequences is one of the key parts of bioinformatics analyses. Resulting phylogenetic trees are beneficial for solving many research questions, including tracing the history of species, studying migration in the past, or tracing a source of a virus outbreak. At the same time, biologists provide more data in the raw form of reads or only on contig-level assembly. Therefore, tools that are able to process those data without supervision need to be developed.

**Results:**

In this paper, we present a tool for reference-free phylogeny capable of handling data where no mature-level assembly is available. The tool allows distance calculation for raw reads, contigs, and the combination of the latter. The tool provides an estimation of the Levenshtein distance between the sequences, which in turn estimates the number of mutations between the organisms. Compared to the previous research, the novelty of the method lies in a newly proposed combination of the read and contig measures, a new method for read-contig mapping, and an efficient embedding of contigs.

**Supplementary Information:**

The online version contains supplementary material available at 10.1186/s13040-023-00329-x.

## Introduction

The genetic code includes not only information about current organisms and their state, but it also contains enough information to trace the history of evolution. With *phylogenetic trees* that represent hypothetical evolutionary trees, one can, for example, trace the migration in the Middle East area [10], find a source of a virus outbreak [9], or solve a hundred-year-old argument between biologists, on the one hand claiming that a panda is a bear and biologists, on the other hand classifying panda as a raccoon [22].

Historically, biologists had to rely on phenotype to build such a tree. With advances in genome sequencing, DNA—the genuine source of differences between species—is used instead. Given a set of DNA sequences, their pairwise similarities can be computed through sequence *alignment*, enabling a subsequent construction of a phylogenetic tree through a hierarchical clustering algorithm.

However, obtaining the needed sequences from biological material is not straightforward. The DNA molecule is first cloned and cut into many short fragments. Then, we select fragments of a similar length, which are sequenced in parallel. The sequenced fragments are called *reads* and the *read length* length is usually between tens to hundreds of nucleotides. Since the read positions in the sequence are unknown, the in-silico *assembly* of the original sequence is based on detected overlaps between input read pairs. Typically, the sequence cannot be reconstructed entirely due to a part of it not being covered by enough reads, the presence of long repeat regions, or other reasons. As a result, instead of the original sequence, the algorithm produces a set of sequence’s non-overlapping substrings called *contigs*.

Additional wet-lab sequencing work is needed to move from contigs toward the target sequence. Usually, so-called mate-paired reads are sequenced. Those are two reads sequenced from the ends of a fragment of a known length. For some mate-pairs, the sequenced ends of the fragments can hopefully be located in two different contigs, which allows building a *scaffold* to order contigs and estimate gaps between them. Then a computationally expensive iterative procedure called *gap-filling* tries to align reads to the ends of the contigs to extend them.

The mentioned sequencing issues are avoided by the *alignment-free approaches* (AF) [39] that estimate the similarities from constant-length subsequences, called *k*-mers, which the input sequences are broken into. This principle naturally allows using raw reads on the input. The main advantage of the AF approaches is that they are, by order of magnitude, faster than the conventional sequence alignment methods [39]. A disadvantage is the obvious loss of information incurred by the breakage into *k*-mers. Indeed, the most promising area for the alignment-based approaches is when the alignment is applied to short, mutually similar sequences [39].

With decreasing costs of genome sequencing, more and more data are being produced, and researchers commonly publish their data in the form of read sets accompanied by their partial assemblies, i.e., with a set of contigs. Here we contribute a method and a tool suitable for said kind of input data leveraging the assembled contigs but also all leftover reads not included in the contigs.

The method combines the advantages of conventional and AF approaches. The method allows distance estimation without the need to determine the complete sequences or to break the already assembled contigs into *k*-mers. Instead, the *k*-mers are used only to select prospective reads and contigs to apply alignment calculation on. In this way, the distance is evaluated on closely matching regions of the sequences. The proposed method does not assume the availability of any reference genome. By avoiding *de novo assembly*, the method requires smaller coverage than the conventional assemble-then-align approach. Compared to the AF methods, the proposed method has a more straightforward connection to the evolutionary distance between the organisms as its goal is to estimate directly the number of point mutations that occurred between the species over time. While the presented approach is slower than the AF methods, the distances calculated by our method are closer to the reference solutions, and the results are usable in more cases.

This study uses some ingredients from our previous work. In particular, we follow up on the method [26, 28] that assumed the inputs to consist exclusively of reads and another one assuming only contigs on the input [27]. In contrast to these previous studies, the present proposal exploits both kinds of inputs synergically. The sequence similarity computation is based on matching reads and contigs from the two input sequences yielding four different matching cases. For read-read matching, we use the algorithm from [26]. For contig-contig matching, we adopt and improve the method [27]. The original method featured favorable accuracies of the computed estimates; however, the quadratic time complexity hindered its practical use. Here we provide an improvement of the technique achieving a speedup by order of one or two magnitudes without significant influence on the quality of the estimates (Efficient contig-contig matching section). Read-to-contig and contig-to-reads matching strategies are novel contributions of this paper (Read-contig mapping, Contig-reads mapping, and Efficient read-contig matching sections).

The salient contribution of this paper is the integration of the four mentioned facets to provide the desired distance estimate (Avoiding redundancy and Combination of the measures sections). We present a robust method that leverages the assembled contigs of the input sequences, but when such contigs are not available, or they cover only a small part of the sequences, the method maintains good accuracy due to its ability to exploit raw reads as well.

## Problem formalization

Here we formalize the problem tackled in the rest of the paper. Table 1 summarizes the notation of the main concepts.

A *string* is a sequence of symbols chosen from $\{\mathsf{a,g,c,t}\}$. As we will not be concerned with sequences other than strings, the words *string* and *sequence* will be used interchangeably. The empty string is denoted as $\varepsilon$, |*x*| means the length of string *x*, and *xy* is the concatenation of strings *x* and *y*. If $x=ps$, then *p* is a *prefix* of string *x*, and *s* is a suffix of it. If $x=pys$, then (*p*, *s*) is an *occurrence of string* *y* *in string* *x*. String *y* is a *substring* of string *x* if there is an occurrence of *y* in *x*. Two strings *overlap* if a non-empty prefix of one is a suffix of the other one. Two substrings *x*, *y* of *z* *overlap in* *z* if there exist strings *u*, *v*, *w* such that *v* is not empty, *uvw* is a substring of *z*, and $x=uv, y=vw$ or $y=uv$, $x=vw$. So, e.g., $\mathsf{ct}$ and $\mathsf{tg}$ overlap in $\mathsf{ctg}$ but not in $\mathsf{ctatg}$.

Given a set of strings $S = \{s_1, s_2, \ldots , s_n\}$, string *s* is a *superstring* of *S* if all $s_i$ ($1 \le i \le n$) are substrings of *s*, and given also $K \in \mathbb {N}$, we define the *bounded superstring problem* followingly: is there a superstring *s* of *S* such that $|s| \le K$? This problem is NP-hard [8].

*Read* *a* of sequence *A* is a short ($|a| \ll |A|$) substring of *A*. *Read bag* $R_{A}$ of *A* is a bag of reads of constant length $l \in \mathbb {N}$ sampled i.i.d. with replacement from the uniform distribution on all possible $|A| - l - 1$ substrings of *A* of length *l*. *Coverage*

1 $$\begin{aligned} c = \frac{l|R_{A}|}{|A|} \end{aligned}$$

indicates the average number of reads covering a particular position in *A*. Here, we assume a constant coverage for all read bags considered.

*Contig* $\alpha$ of sequence *A* is a substring of *A*, and *contig set* $C_{A}$ of *A* is a set of contigs of *A* such that no two contigs in $C_{A}$ overlap in *A*.^1^

The *sequence assembly* task is to reconstruct sequence *A* from its read bag $R_{A}$. This general task statement does not allow any guarantees for an exact solution; one cannot be, for example, sure that reads in $R_{A}$ cover all positions in *A*. Typically, a surrogate problem is, therefore, considered instead: find the shortest superstring of $R_{A}$. While this formulation has a well-defined solution, it is, of course, at least as hard as the bounded superstring problem and, thus, NP-hard. As such, it is usually tackled in a heuristic manner by the iterative merging of overlapping read pairs, generally resulting in multiple mutually disconnected assembled sequences (contigs) and a bag of remaining reads not used in the contigs.^2^

Let $\mathsf{dist}(A, B)$ denote the *Levenshtein distance* [16] between sequences *A* and *B*, i.e., the minimum number of operations *insert*, *delete*, and *substitute* needed to make the two sequences the same.^3^

**Table 1:** An overview of the notation used in the paper

symbols	meaning
*A*, *B*	genomic sequences
$R_{A}, R_{A}'$ ($R_{B},R_{B}'$)	read bags sequenced from *A* (*B*)
*a*, *b*	a read from $R_{A}$ ($R_{B}$)
$C_{A}$ ($C_{B}$)	contig set for *A* (*B*) assembled from $R_{A}$ ($R_{B}$)
$\alpha$ ($\beta$)	a contig from $C_{A}$ ($C_{B}$)
$\alpha ^{*}$ ($\beta ^{*}$)	a substring of contig $\alpha$ ($\beta$)
$T_{A}$ ($T_{B}$)	$R_{A}, C_{A}$ ($R_{B}, C_{B}$) tuple
$\mathsf{dist}(\cdot ,\cdot )$	Levenshtein distance function
$\mathsf{dist}_{xy}(\cdot ,\cdot )$	auxiliary (non-Levenshtein) distance functions, *x*, *y* indicate the respective argument types: $\mathsf{r}$ - read, $\mathsf{c}$ - contig, $\mathsf{R}$ - read bag, $\mathsf{C}$ - contig set, $\mathsf{T}$ - read-contig tuple, $\mathsf{T}\backslash \mathsf{R}$ -

The task we deal with in this paper is to estimate $\mathsf{dist}(A, B)$ given contig set $C_{A}$ and read bag $R_{A}$ of *A*, and the analogical inputs $C_{B}, R_{B}$ for *B*. Again, stated this way, the problem does not provide any guarantees for the exact solution for reasons including incomplete coverage by reads, but also the fact that the sum of lengths of the input contigs may be larger than the original sequence length. Analogically to the sequence assembly task, we consider instead a surrogate problem defined in turn.

Given two contig sets, $C_{A}, C_{B}$, and two read bags, $R_{A}, R_{B}$, the *partially assembled sequences distance problem* (PASDP) is to determine $\mathsf{dist}(\tilde{A}, \tilde{B})$ where $\tilde{A}$ ($\tilde{B}$, respectively) is the shortest superstring of $R_{A} \cup C_{A}$ ($R_{B} \cup C_{B}$).

We now show that PASDP is NP-hard. Let (*S*, *K*) be an instance of the bounded superstring problem. We will show that it reduces to PASDP. Let $C_{A} = C_{B} = \emptyset$. Let $R_{A} = S$ and $R_{B} = \emptyset$. Then $\tilde{B} = \varepsilon$ and $\tilde{A}$ is the shortest superstring of *S*. As $\tilde{B}$ is empty, by the definition of the Levenshtein distance,

2 $$\begin{aligned} \mathsf{dist}(\tilde{A}, \tilde{B}) = | \tilde{A} | = | \text {shortest superstring of}\ S|. \end{aligned}$$

The bounded superstring problem is answered positively if and only if $\mathsf{dist}(\tilde{A}, \tilde{B}) \le K$. Therefore, PASDP is at least as hard as the bounded superstring problem and thus is indeed NP-hard.

## Related work

This research relates to *alignment-free measures* (AF measures). For sequence *A*, we can define the *q-gram profile* as vector $\textbf{Q}^{A}_{k}$ whose *i*-th component is equal to the number of occurrences of the *i*-th *q*-gram^4^ in *A*. The first AF measure, $D_2$, was proposed by [4] as simply the scalar product of $\textbf{Q}^{A}_{k}$ and $\textbf{Q}_{k}^{B}$. Many other measures followed, including, $D_2^S$, *D*2*z*, [13, 25], differing mostly only in normalization or bias removal to capture the real nature of the data. A recent overview of the AF methods is in [39].

We will compare our approach with two state-of-the-art algorithms namely *co-phylog* [37], and *Mash* [23].

The co-phylog algorithm introduces a notation of *C*-gram subsequences. Each *C*-gram has one or more positions called *O*-grams. On *C*-gram positions, an exact match is required. On *O*-gram positions, any nucleotide is acceptable. For both sequences, a mapping is created from *C*-grams to corresponding *O*-grams. This mapping is then used to calculate the distance as similar sequences have a higher number of shared *C*-gram-*O*-gram pairs.

The Mash algorithm is based on the hashing approach used originally for embedding. In its nature, Mash is very similar to the $D_2$ measure. For each *k*-mer of the sequence, its hash is calculated. It is not guaranteed that two distinct *k*-mers have different hashes; however, this situation is not very likely-the algorithm then computes the Jaccard index of the set of hashes. The main advantage of the algorithm compared to the $D_2$ measure is its effectiveness, as it is much easier to store the set of hashes in the memory than the whole set of *k*-mers.

The success of the alignment-free tools justifies the need for alignment-free and assembly-free tools. Review paper [39] mentioned more than 100 tools published in 2017. The Mash tool meanwhile collected more than 1, 000 citations according to the publisher’s website.

## Proposed method

The non-tractability of PASDP motivates a heuristic approach to compute a sub-optimal solution. To this end, we avoid any attempts to reconstruct *A* and *B* from the input reads and contigs, as these inputs are already assumed to be the ultimate outputs of an assembly algorithm so further overlap-based assembly is futile.

To estimate the distance between the sequences, we will be matching strings (reads and contigs) from one sequence with those of the other. Each such match will yield a contribution to the final *distance* (dissimilarity function). We follow the “Occam’s razor” heuristic that for a string from one sequence, we should look for the closest match in the other sequence to yield the distance contribution. This rationale is supported by the fact that if the original sequences were known, their dissimilarity would be computed under their best possible alignment.

**Fig. 1:**
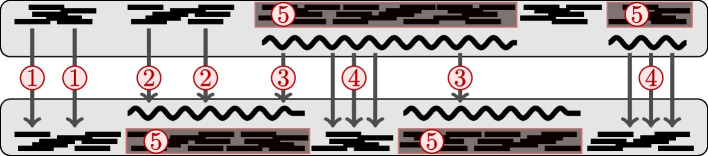
An overview of possible mappings between reads and contigs. A read can map either to a read (①, [28]) or a contig (②, Read-contig mapping section). Similarly, a contig can map to a part of another contig (③, [27]) or multiple reads (④, Contig-reads mapping section). Reads that were assembled to a contig do not need to be considered (⑤, Avoiding redundancy section)

**Fig. 2:**
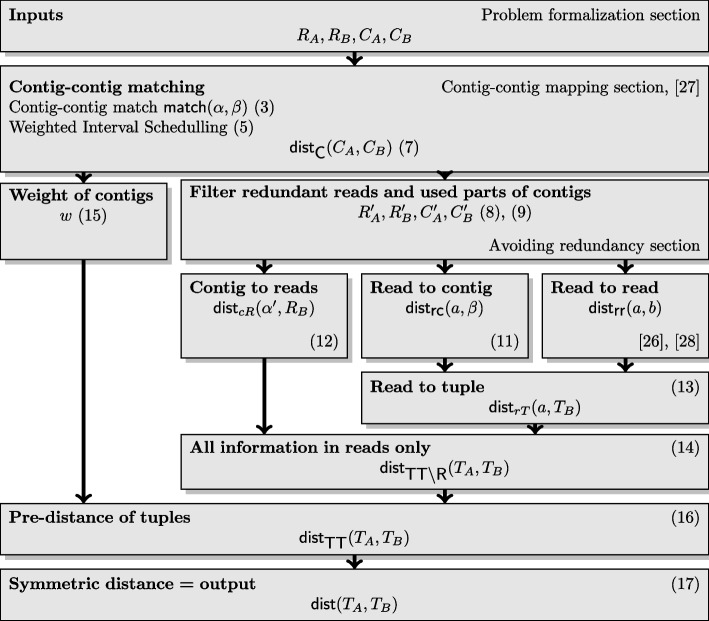
An overview of the algorithm with references to the corresponding sections and equations

The matching strategy differs for the four possible pairing sorts (refer to Fig. 1): read-to-read, read-to-contig, contig to one or more reads, and contig to one or more contigs. This section details them individually and also describes how the matching results integrate into the final distance estimate. Fig. 2 reveals the data flow among the methodological components.

### Contig-contig mapping

Here we explain how we match two contigs by identifying a substring in each of them such that the two substrings have a small Levenshtein distance and also are sufficiently long. Then we elaborate on a matching between a contig and a contig set based on a dynamic programming procedure using the results of the contig-contig matches. Finally, we define the match between two contig sets.

There are two ways in which two contigs can be matched: 1) one is an (approximate) substring of the other, or 2) the two (approximately) overlap. We seek a match that is as long as possible, and at the same time, the distance of the matched parts should be as small as possible. This results in the minimization of the *post-normalized Levenshtein distance*, which is the ratio of the Levenshtein distance and the maximum of the sequence lengths. Formally, we define the match of two contigs $\alpha \in C_{A}, \beta \in C_{B}$ as

3 $$\begin{aligned} \mathsf{match}(\alpha ,\beta ) = \underset{(\alpha ^{*},\beta ^{*}) \in S(\alpha ,\beta )}{\mathsf {arg\,min}} \frac{\mathsf{dist}(\alpha ^{*},\beta ^{*})}{\max \{ |\alpha ^{*}|, |\beta ^{*}| \}}, \end{aligned}$$

where

4 $$\begin{aligned} S(\alpha , \beta ) = \mathsf{suff}(\alpha ) \times \mathsf{pref}(\beta ) \cup \mathsf{pref}(\alpha ) \times \mathsf{suff}(\beta ) \cup \mathsf{sub}(\alpha ) \times \{ \beta \} \cup \{ \alpha \} \times \mathsf{sub}(\beta ). \end{aligned}$$

By symbols $\mathsf{pref}$, $\mathsf{suff}$, and $\mathsf{sub}$, we mean the set of all non-empty prefixes, suffixes, and substrings, respectively. To avoid small random overlaps, a threshold^5^ of 20 is applied on lengths $|\alpha ^{*}|, |\beta ^{*}|$.

For each contig $\alpha \in C_{A}$, we can calculate the match with each contig in $C_{B}$. As contigs represent non-overlapping subsequences of the genome, each symbol of $\alpha$ should be mapped to at most one contig from $C_{B}$. For each $\alpha$, we thus identify a set of contigs in $C_{B}$ that satisfies the said condition. This is achieved by a reduction to the *weighted interval scheduling problem* [14]. The latter is solved by an efficient dynamic-programming procedure (see [27] for details) and yields an admissible subset of matches for contig $\alpha$:

5 $$\begin{aligned} \mathsf{match}(\alpha , C_{B}) \subseteq \{ \mathsf{match}(\alpha , \beta ) \mid \beta \in C_{B} \}. \end{aligned}$$

Next, we define the set of all admissible matches for $C_{A}$:

6 $$\begin{aligned} \mathsf{match}(C_{A}, C_{B}) = \bigcup _{\alpha \in C_{A}} \mathsf{match}(\alpha , C_{B}). \end{aligned}$$

To calculate the distance between $C_{A}$ and $C_{B}$, we sum the distances of the string pairs in $\mathsf{match}(C_{A}, C_{B})$ (the nominator in (7)). Because the overlap lengths do not necessarily correlate with sequence lengths, the measure is normalized^6^ (the denominator in Eq. (7)):

7 $$\begin{aligned} \mathsf{dist}_{\mathsf{C}}(C_{A}, C_{B}) = \frac{\sum _{(\alpha ^{*},\beta ^{*}) \in \mathsf{match}(C_{A}, C_{B})} \mathsf{dist}(\alpha ^{*},\beta ^{*})}{\sum _{(\alpha ^{*},\beta ^{*}) \in \mathsf{match}(C_{A}, C_{B})} \max \{ |\alpha ^{*}|, |\beta ^{*}|\}} . \end{aligned}$$

### Avoiding redundancy

Before we start with mapping reads to contigs, we need to filter out duplicate information. There are two reasons for that — runtime and the aim to avoid bias caused by reusing some parts of sequences twice. Firstly, contigs are generated from reads. There is no need to find matches for a read assembled into a contig since we already found a match for the contig.

To avoid this duplicate work, we re-define

8 $$\begin{aligned} R_{A}' \leftarrow R_{A} \setminus \{ i \in R_{A} \mid i\ \text {is a substring of some}\ \alpha \in C_{A} \}. \end{aligned}$$

To eliminate the reads that are substrings of a contig, we use the Aho-Corasick algorithm [2], which builds an automaton on reads in $R_{A}$ and finds their occurrences in a single linear pass through a contig.

Secondly, if a part of contig $\alpha \in C_{A}$ is matched with another contig in $C_{B}$, we do not need to match it with reads in $R_{B}$ — we know that the counterparts are already in $C_{B}$. Instead of $C_{A}$, we work further with $C_{A}'$ that contains for all $\alpha$ only substrings of $\alpha$ that are not in $\mathsf{match}(\alpha , C_{B})$. In other words, let $\mathsf{match}(\alpha , C_{B}) = \{ (\alpha ^{*}_1, \beta _1), (\alpha ^{*}_2, \beta _2), \ldots , (\alpha ^{*}_n, \beta _n) \}$, let $\alpha = \alpha _0' \alpha ^{*}_1 \alpha _1' \alpha ^{*}_2 \alpha _2' \cdots \alpha ^{*}_n \alpha _n'$, and let

$$\begin{aligned} \overline{\mathsf{match}}(\alpha , C_{B}) = \{ \alpha _0', \alpha _1', \alpha _2' \ldots \alpha _n' \} \end{aligned}$$

be a set of all substrings of $\alpha$ that are not matched to any contig in $C_{B}$. Then,

9 $$\begin{aligned} C_{A}' = \bigcup _{\alpha \in C_{A}} \overline{\mathsf{match}}(\alpha , C_{B}). \end{aligned}$$

### Read-read mapping

We adopt the method from [28] based on the Monge-Elkan distance [18] to establish the distance function for two read bags. It follows the same spirit as above; in particular, each read *a* in $R_{A}$ is matched with the closest read in $R_{B}$. Formally, the Monge-Elkan distance is defined as

10 $$\begin{aligned} \underset{\mathsf{ME}}{\mathsf{dist}} (R_{A}, R_{B}) = \frac{1}{|R_{A}|} \sum _{a \in R_{A}} \min _{b \in R_{B}} \mathsf{dist}_{\mathsf{rr}} (a, b) . \end{aligned}$$

The read-read distance $\mathsf{dist}_{\mathsf{rr}}$ above is essentially the Levenshtein distance except for the following adjustment. Because read locations are random, the first $t = \frac{1}{2} \left( \frac{l}{c} - 1 \right)$ leading or trailing gaps are not penalized in the alignment of two reads.^7^

The range of $\mathsf{dist}_{\mathsf{ME}}$ is [0; *l*] as the function is the average of values in the said interval. On the contrary, the Levenshtein distance is in the range of $[0; \max \{|A|, |B|\}]$. In the experiments, we will use the scaled symmetric version of the distance, denoted $\mathsf{dist}_{\mathsf{MESSG}}$.

### Read-contig mapping

When aligning a read to a contig, the read can either overlap with one of the contig ends, or it can match a substring of the contig. Therefore, the match for the read can be defined as a substring of $\beta$ that has the lowest distance from *a* but for borders where the first *t* margin gaps are not penalized. Otherwise, the read-contig distance is defined as

11 $$\begin{aligned} \mathsf{dist}_{\mathsf{rc}}(a, \beta ) = \min _{\begin{array}{c} i \in [2, |\beta |-l-2], \\ j \in [i+1, |\beta |-l-1] \end{array}} \mathsf{dist}(a, \beta _i^{j}), \end{aligned}$$

where $\beta _i^{j}$ denotes a substring of $\beta$ that starts at *i* and ends at *j*.

**Fig. 3:**
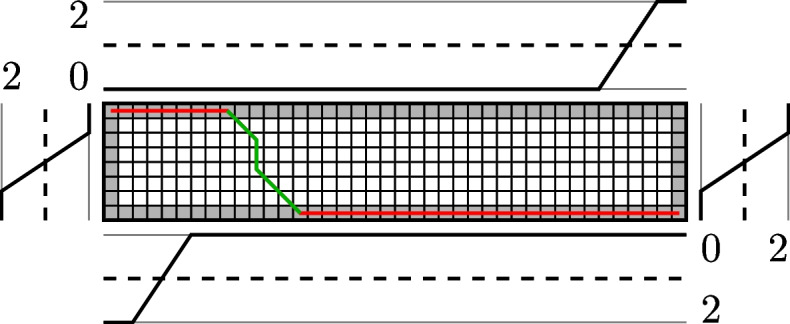
An illustration to (11). Instead of constant 1 (dashed line), the gap extension penalty on margins changes to the solid line. In this case, the cost-free margin gaps are *t* = 2

Following the reasoning from Read-read mapping section, it is not desirable to penalize leading or trailing gaps up to a certain length at contig ends. Therefore, we modify the Wagner-Fischer dynamic programming algorithm [34] so that it does not penalize the first *t* leading or trailing gaps caused by a random location of the read or different lengths of the contig and the read. The cost function used for the margin gap penalty is illustrated in Fig. 3.

### Contig-reads mapping

Here, the mapping is very similar to the one used in the previous section. There is, however, a slight difference — contig $\alpha$ is long, and as a result, there should be multiple reads $b \in R_{B}$ that map to $\alpha$. By (1), there should be $|\alpha | \cdot c / l$ reads on average. Therefore, we calculate the $\mathsf{dist}_{\mathsf{rc}}$ distance from $\alpha$ to all $b \in R_{B}$ and select the $|\alpha | \cdot c / l$ minimum distances.

From Avoiding redundancy section, we know that we will use only unmatched substrings $\alpha '$. Therefore, we define dissimilarity for $\alpha '$ as

12 $$\begin{aligned} \underset{\mathsf{cR}}{\mathsf{dist}}(\alpha ', R_{B}) = \min _{\left\{ S \subseteq R_{B}' \left| |S| = \left\lfloor \frac{|\alpha '| \cdot c}{l} \right\rfloor \right. \right\} }\sum _{b \in S} \mathsf{dist}_{\mathsf{rc}} (b, \alpha '). \end{aligned}$$

The formula above selects subset *S* of read bag $R_{B}'$ that minimizes the sum of the distances between the reads and $\alpha '$.

### Combination of the measures

The final measure based on reads follows Eq. (10). For each read $a' \in R_{A}'$, there should be exactly one match — either a read or a contig. We define the distance from a read to the closest read or contig as

13 $$\begin{aligned} \underset{\mathsf{rT}}{\mathsf{dist}}(a', T_{B}) = \min \left\{ \min _{b' \in R_{B}'} \mathsf{dist}_{\mathsf{rr}}(a', b'), \min _{\beta \in C_{B}} \mathsf{dist}_{\mathsf{rc}}(a', \beta ) \right\} . \end{aligned}$$

The sum of (13) and (12) over all reads and contigs gives a pre-measure that contains all information captured in reads as

14 $$\begin{aligned} \mathsf{dist}_{\mathsf{TT}\backslash \mathsf{R}}(T_{A}, T_{B}) = \frac{\displaystyle \sum _{a' \in R_{A}'} \mathsf{dist}_{\mathsf{rT}}(a', T_{B}) + \sum _{\alpha ' \in C_{A}'} \mathsf{dist}_{\mathsf{cR}}(\alpha ', R_{B})}{l \left( |R_{A}'| + \sum _{\alpha ' \in C_{A}'} \left\lfloor \frac{|\alpha '| \cdot c}{l} \right\rfloor \right) }. \end{aligned}$$

The denominator in Eq. (14) scales to [0, 1] interval and is calculated by substituting *l* for distance calculations.

In the next step, we will have to combine $\mathsf{dist}_{\mathsf{TT}\backslash \mathsf{R}}(T_{A}, T_{B})$ with the measure capturing the distance between the contigs. To do so, we use a weighted average based on the part of the original sequences covered by the contigs. $\mathsf{dist}_{\mathsf{C}}(C_{A}, C_{B})$ uses only a part of sequence *A*, namely

$$\begin{aligned} |\mathsf{match}|(C_{A}, C_{B}) = \sum _{(\alpha ^{*}, \cdot ) \in \mathsf{match}(C_{A}, C_{B})} |\alpha ^{*}|. \end{aligned}$$

On the contrary, the estimate of *A* length is $|R_{A}| \cdot l / c$. The weight assigned to the contig-contig measure is, therefore,

15 $$\begin{aligned} w = \min \left\{ 1, |\mathsf{match}|(C_{A}, C_{B}) \cdot \frac{c}{|R_{A}| l}\right\} . \end{aligned}$$

The $\min$ operation is to prevent errors of assembly because there is no guarantee that the contigs will be shorter than the true sequence. We define pre-distance as

16 $$\begin{aligned} \mathsf{dist}_{\mathsf{TT}}(T_{A}, T_{B}) = w \mathsf{dist}_{\mathsf{C}}(C_{A}, C_{B}) + (1-w) \mathsf{dist}_{\mathsf{TT}\backslash \mathsf{R}}(T_{A}, T_{B}) . \end{aligned}$$

The final distance is the scaled (see Formula (1) — $\mathsf{dist}_{\mathsf{TT}}$ is from the [0, 1] interval while $\mathsf{dist}(A, B)$ is from the $[0, \max \{|A|, |B|\}]$ interval) symmetric version

17 $$\begin{aligned} {\mathsf{dist}}(T_{A}, T_{B}) = \frac{\mathsf{dist}_{\mathsf{TT}}(T_{A}, T_{B}) + \mathsf{dist}_{\mathsf{TT}}(T_{B}, T_{A})}{2} \cdot \frac{l \cdot \max \{|R_{A}|, |R_{B}|\}}{c} . \end{aligned}$$

## Efficiency improvements

**Fig. 4:**
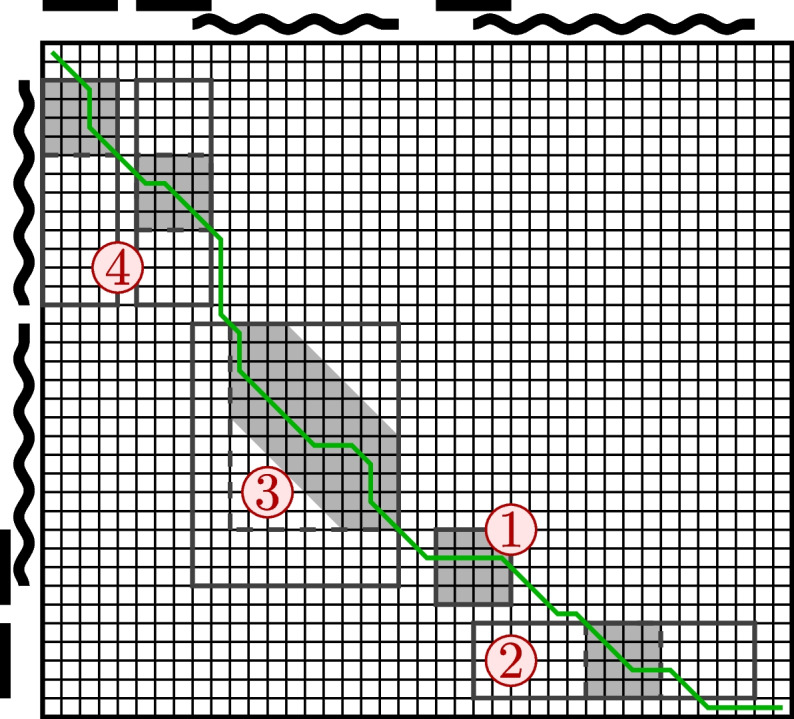
The main idea of the efficiency improvements in Efficiency improvements section. Instead of pairwise alignment of all reads and contigs vs. all reads and contigs, we run the exact quadratic alignment only for a small subset of those. This subset of candidates is identified based on the *q*-gram distance, which runs in linear time. See Fig. 1 for meaning of the caption numbers

Equation (17) gives a way to compute the distance. However, this method is too slow for practical use as it requires the alignment of all data in $T_{A}$ and $T_{B}$. Most of those alignments are, however, not necessary as they do not count towards the minimum in (5), (10), and (12). Therefore, we will use only a carefully selected subset of alignments, as illustrated in Fig. 4. Following [26], we use the technique of embedding reads to *q-grams* to filter out alignments that won’t count towards the output of the $\min$ operator. Recall the *q-gram profile* $\textbf{Q}^{A}_{k}$ of a string *A* defined in Related work section. The *q-gram distance* of *A* and *B*, denoted $\mathsf{dist}_q (A,B)$, is the Manhattan distance of *q*-gram profiles of *A* and *B*, i.e., $\mathsf{dist}_q(A,B) = \Vert \textbf{Q}^{A}_{k} - \textbf{Q}_{k}^{B} \Vert _1$.

Informally, the *q*-gram distance counts how many substrings of length *q* are in one sequence and not in the other (or vice versa). The *q*-gram distance is a simple but effective approximation of the Levenshtein distance [33] as it holds that

18 $$\begin{aligned} \mathsf{dist} (A, B) \ge \frac{1}{2q} \cdot \mathsf{dist}_q (A, B). \end{aligned}$$

While we need $\mathcal {O}(|A||B|)$ time to calculate the Levenshtein distance, the *q*-gram distance is much faster to compute: we need $\mathcal {O}(q + |A| + |B|) + 4^q)$ or only $\mathcal {O}(4^{q})$ if we precompute the *q*-gram profiles in advance.

### Efficient read-read matching

Calculating the edit distance between two reads with the Wagner-Fischer algorithm [34] (called Needleman-Wunsch [20] in the bioinformatics context) is very fast as reads are usually only hundreds of symbols long. However, the Monge-Elkan distance requires us to calculate the distance between all pairs of reads. We, however, need only the closest match in Eq. (10). Therefore, as a heuristic, we select a candidate for the match (there may be more of them than one) under the *q*-gram distance for each read. Because of the typical read length, we use $q=3$. Once having only a few closest match candidates, we can calculate the exact Levenshtein distance.

### Efficient read-contig matching

Matching a read to a contig means finding the contig’s substring, which is the most similar to the read. Instead of the quadratic dynamic programming approach, we can exploit the *q*-gram distance and find a set of candidates in linear time. Then for this short substring, we can call the exact quadratic alignment.

A naive implementation of the search under the *q*-gram distance requires a quadratic amount of work. This can be cut down to linear time using the sliding window method. Once we have calculated the *q*-gram distance for a prefix of length *l* of the contig, we can only update the distance by observing the first-to-add *q*-gram of the contig and the last-to-remove *q*-gram of the contig. Once we have the correct candidates, we trigger the exact quadratic alignment.

### Efficient contig-contig matching

We relax the matching problem by positing the following assumption. Referring to (3), we assume that for $(\alpha ^{*}, \beta ^{*}) = \mathsf{match}(\alpha , \beta )$, it holds that $|\alpha ^{*}| = |\beta ^{*}|$. This reduces the quadratic number of possible overlap candidates to a linear number.

We adapt the sliding window approach from the previous section that updates the current distance only by two *q*-grams as contigs slide one against each other. This allows us to find a match candidate that minimizes the ratio of the *q*-gram distance over the length of the match. Once having this candidate, we can calculate the exact Levenshtein distance of the matching contig parts faster using Ukkonen’s cutoff heuristic from [32] and [3].

We now address the choice of *q*. In the two preceding sections, we compared sequences of fixed length *l*. Here, the overlap length grows up to the minimum of the contig lengths. According to study [19], the *q*-gram distance works well for sequences of length $4^q$. With longer sequences, the *q*-gram profiles start to get closer to a fixed distribution (uniform for random sequences). To avoid this bias towards the long overlaps, we need to switch between *q* values as overlaps get longer. For a value of *q*, we consider overlaps of length from the interval $[4^{q-1}, 4^{q+1}]$. However, direct comparison is still not possible, as our goal is to minimize the Levenshtein distance. Instead of comparing the ratio of $\mathsf{dist}_q$ over the overlap length, we divide this ratio by *q*, which is a direct result of (18).

## Experiments

To evaluate the method, we run the algorithm on several real-world and simulated datasets. The simulated data will allow us to study the method under a wide range of coverage and read length, which is not available for real-world data.

### Tested algorithms

The tested algorithms can be divided into several groups. The first group includes *trivial baselines* that should illustrate the complexity of the problem. This includes $\max \{|R_{A}|, |R_{B}|\}$, a simple upper bound on the Levenshtein distance, and the Levenshtein distance between the two longest contigs as produced by an assembly algorithm. Next, the comparison includes our previous work (distances $\mathsf{dist}_{MESSG}$ [28], $\mathsf{dist}_{MESSGq}$ [26], $\mathsf{dist}_{\mathsf{C}}$ [27]), the newly proposed method $\mathsf{dist}$ (as of Eq. (17)), and $\mathsf{dist}_q$ (with improvements from Efficiency improvements section, alternatively including downsampling denoted by $\alpha$ index). The state-of-the-art alignment-free approaches include Mash [23], *d*-type measures [5], and co-phylog [37]. The alignment-free approaches were initialized with the default set of parameters, and the *d*-type measures used $k=5$. The alignment-free measures used only reads as input.

Any time contigs were used as an input in one of the methods, we used contigs calculated from the aforementioned read bags together with five *assembly algorithms*, namely ABySS [30], Edena [11], SPADES [21], SSAKE [35], and Velvet [38]. All assembly algorithms were initialized with the default parameters whenever possible.

### Used datasets

The *influenza* dataset consists of 13 real-world virus DNA sequences. Reads from those sequences are then sampled uniformly under the i.i.d. assumption. The sampling of reads was done for 20 coverage values in a range from 0.1 to 100. We used 14 read length values in a range from 3 to 500. The original sequences were used as a reference for distance calculation using the Wagner-Fischer algorithm [34]. The sequences were downloaded from the ENA repository [15] and were selected to contain similar virus genomes. The *various* dataset contains sequences of different viruses and undergoes the same procedure. The *hepatitis* dataset contains 81 *hepatitis* sequences. This time, the read sampling was done with the ART program [12] for $(\alpha , l) \in \{10, 30, 50\} \times \{30, 70, 100\}$.

We use two real-world datasets. The first one contains sequences that are supposed to be completely different. We selected $20\,\text {kbp}$ regions from the human DNA, each region from the beginning of one chromosome (excluding telomeres). For those regions, we selected reads that map to those regions. The reads come from the 1000 genome project [1]. The second real-world dataset, the *e-coli* dataset, contains 15 bacterial DNA of the same species. In this case, no official assembly for each of the read bags exists; therefore, we compare the algorithms in quantitative measures that do not need assembly.

### Evaluation criteria

We evaluate the method in both qualitative as well as quantitative measures. The qualitative measures are represented by *Pearson’s correlation coefficient* and the Fowlkes-Mallows index [7]. The first measure compares the similarity of the distance matrices by calculating the correlation coefficient on the distances to the reference distances calculated from the original sequences using the Wagner-Fischer algorithm [34].

The *Fowlkes-Mallows index* $B_k$ is used to compare the resulting phylogenetic trees built by the neighbor-joining algorithm [29]. To calculate $B_k$, the tree is cut at level *k* to obtain several clusters. For the tested method and the reference, the clusterings are then compared. Besides the Fowlkes-Mallows index, the second method used to compare the trees is the *triplets distance* [6]. The distance counts how many times a set of three organisms form a tree of different topology.

The quantitative measures compare how well the method performs in terms of time and how often the method produces a valid result (for low coverage, an assembly algorithm may fail to produce any contigs, disqualifying all methods that depend only on contigs or calculation tool longer than the time limit of two hours (1 day on the hepatitis dataset)). Besides the *finished* cases, we measure the *assembly time* and the *distance matrix time* separately.

**Table 2:** Summary of the results. The finished column shows how many times the distance calculation was successful for different choices of read length and correlation. The following columns contain average assembly time, distance matrix calculation time, Pearson’s correlation coefficient of distance matrices, the Fowlkes-Mallows index for $k=4$ and $k=8$, and the triplets distance. Note that the triplets distance is calculated only on a sample of read length and coverage values on the influenza and various datasets. The averaged results are only for the situations when the method finished. The rank columns show the average rank of the methods in distance calculation time and correlation, including the situations when the method did not finish. The ‘reference’ method calculates distances of the original sequences. We show only assembly algorithms that gave the highest and the lowest correlation. From *d*-type measures, the one with the highest correlation is selected. For an explanation of the rank column, see Evaluation criteria section

$\mathsf{Data}$	$\mathsf{method}$	$\mathsf{finished}$	$\frac{\mathsf {assem.}}{\textrm{ms}}$	$\frac{\mathsf{distances}}{\textrm{ms}}$	$\frac{\mathsf{rank}}{\mathsf{distances}}$	$\mathsf {corr.}$	$\frac{\mathsf{rank}}{\mathsf {corr.}}$	$B_{4}$	$B_{8}$	$\mathsf {trip.d.}$
$\mathsf{Influenza}$	$\mathsf{reference}$	112/112	0	2,602	29.2	1	1	1	1	0
	max(|$R_{A}$|,|$R_{B}$|)	112/112	0	335	**13.3**	.801	46.5	.66	.32	57
	$\mathsf{dist}_{\mathsf{MESSG}}(R_{A},\ R_{B})$	107/112	0	899,270	60.1	**.983**	**9.7**	1	1	5
	$\mathsf{dist}_{\mathsf{MESSGq}}$	112/112	0	50,808	42.5	.966	27.9	1	.97	28
	$\mathsf{dist}_{\mathsf{C}} \mathsf{SPAdes}$	43/112	13,529	22,661	56.8	.973	49.4	.99	.93	8
	$\mathsf{dist}_{\mathsf{C}} \mathsf{SSAKE}$	68/112	2,079	17,735	48.5	.944	44.5	.97	.84	22
	$\mathsf{dist} \mathsf{SPAdes}$	112/112	12,380	625,883	56.7	**.983**	**8.9**	1	1	0
	$\mathsf{dist} \mathsf{Velvet}$	111/112	378	749,033	57.9	.971	29.1	1	.99	23
	$\mathsf{dist}_{q} \mathsf{SPAdes}$	112/112	14,345	28,690	37.6	.971	23.1	1	.94	28
	$\mathsf{dist}_{q} \mathsf{Velvet}$	112/112	446	22,478	37.7	.956	35.3	1	.97	38
	$\mathsf{Mash}$	112/112	0	**101**	**9**	.679	46.8	.44	.61	152
	$d_{2}^{*}$	112/112	0	389	18.3	.837	44.7	.4	.9	118
	$\mathsf{longest contig SPAdes}$	43/112	13,529	1,465	48.2	.751	51.5	.71	.56	106
	$\mathsf{longest contig Velvet}$	110/112	385	**38**	**7.5**	.569	53.8	.46	.23	133
$\mathsf{Various}$	$\mathsf{reference}$	112/112	0	57,099	16.9	1	1	1	1	0
	$\mathsf{max}(|R_{A}|,|R_{B}|)$	112/112	0	847	**4.1**	.907	14.1	.85	.92	48
	$\mathsf{dist}_{\mathsf{MESSG}}$	64/112	0	1,299,980	24.8	**.933**	13	.93	.93	19
	$\mathsf{dist}_{\mathsf{MESSGq}}$	109/112	0	605,647	20	.927	**8.7**	.84	.97	42
	$\mathsf{dist}_{\mathsf{C}} \mathsf{SSAKE}$	108/112	1,235	749,197	20.7	.928	**5.4**	.84	.92	25
	$\mathsf{dist}_{\mathsf{C}} \mathsf{Velvet}$	34/112	17,783	1,239,632	25.5	.917	19.8	.88	.94	16
	$\mathsf{dist} \mathsf{Edena}$	69/112	168	1,681,308	24.6	**.932**	12.3	.92	.93	18
	$\mathsf{dist} \mathsf{SSAKE}$	64/112	568	1,635,059	26.1	.919	12.9	.83	.91	27
	$\mathsf{dist}_{q} \mathsf{ABySS}$	110/112	10,937	252,197	16.5	.919	11.7	.85	.93	39
	$\mathsf{dist}_{q} \mathsf{SSAKE}$	111/112	2,231	428,540	17.9	**.934**	**6.4**	.84	.95	62
	$\mathsf{Mash}$	84/112	0	**562**	8.3	.664	17.8	.46	.34	344
	$d^{q*}_{2}$	109/112	0	**721**	8	.573	17.4	.32	.28	399
	$\mathsf{longest contig SSAKE}$	108/112	1,235	**385**	**3.5**	.386	20.9	.48	.43	349
	$\mathsf{longest contig Velvet}$	34/112	17,783	34,858	22.5	.681	21.4	.62	.5	329
Hepatitis	reference	9/9	0	1,748,984	16.9	1	1	1	1	0
	max(|$R_{A}$|,|$R_{B}$|)	9/9	0	29,340	5.8	.181	19.3	.72	.83	24,017
	$\mathsf{dist}_{\mathsf{MESSG}}$	9/9	0	42,332,682	21.1	.965	8.3	1	.9	4,407
	$\mathsf{dist}_{\mathsf{MESSGq}\alpha }$	9/9	0	1,118,585	15.4	.897	14.2	1	.94	4,543
	$\mathsf{dist}_{\mathsf{C}} \mathsf{SPAdes}$	2/9	76,514	31,517,537	23.1	.869	20.6	1	.89	7,361
	$\mathsf{dist}_{\mathsf{C}} \mathsf{Velvet}$	4/9	11,090	59,898,794	23.9	**.98**	14.6	1	.99	2,419
	$\mathsf{dist} \mathsf{Edena}$	0/9	NaN	NaN	24.4	NaN	24.4	NaN	NaN	NaN
	$\mathsf{dist}_{q\alpha }\ \mathsf{ABySS}$	9/9	48,194	520,227	12.7	.957	10.6	1	.93	13,051
	$\mathsf{dist}_{q\alpha }\ \mathsf{SSAKE}$	9/9	88,516	615,615	14.2	.901	12.9	.96	.94	13,710
	$\mathsf{Mash}$	9/9	0	**2,350**	**1.4**	.967	8.1	1	.92	9,532
	$d^{q}_{2}$	9/9	0	27,885	6.7	**.973**	**5.1**	1	.87	5,347
	$\mathsf{longest contig Edena}$	9/9	7,038	1,581,613	15.6	.515	17.8	.92	.76	23,452
	$\mathsf{longest contig Velvet}$	4/9	11,090	515	13.1	.296	21.3	.92	.47	51,443
$\mathsf{Chroms}$	$\mathsf{reference}$	1/1	0	668,767	20	1	1	1	1	0
	max(|$R_{A}$|,|$R_{B}$|)	1/1	0	2,184	13	.331	18	.61	.3	880
	$\mathsf{dist}_{\mathsf{MESSG}}$	1/1	0	23,758,416	24	.848	14	.58	.26	923
	$\mathsf{dist}_{\mathsf{MESSGq}\alpha }$	1/1	0	202,517	19	.825	15	.9	.25	939
	$\mathsf{dist} \mathsf{ABySS}$	1/1	17,838	24,085,638	25	.911	6	.64	.34	707
	$\mathsf{dist} \mathsf{SPAdes}$	1/1	22,898	23,757,934	23	.873	13	.68	.21	968
	$\mathsf{dist}_{q\alpha }\ \mathsf{SPAdes}$	1/1	22,898	127,061	16	.881	11	.81	.33	991
	$\mathsf{dist}_{q\alpha }\ \mathsf{SSAKE}$	1/1	51,604	126,565	15	**.914**	**4**	.81	.21	987
	$\mathsf{Mash}$	1/1	0	**173**	**3**	.33	19	.6	.38	787
	$d^{q*}_{2}$	1/1	0	697	6	**.959**	**2**	.81	.32	1,083
	$\mathsf{longest contig Velvet}$	1/1	7,866	31	1	.574	16	.81	.4	1,007

The boldface numbers mark three best results on each dataset

Please note, that some of the marked results might be in the Supplementary materials

**Table 3:** Pairwise correlations of distance matrices on “E. coli” dataset. The last two columns show the runtime on the “E. coli” dataset. Assembly time (without distance matrix calculation) on the same dataset is $24{,}980\,\textrm{s}$ (ABySS), $17{,}514\,\textrm{s}$ (Edena), $1021{,}184\,\textrm{s}$ (SPAdes), $229{,}350\,\textrm{s}$ (SSAKE), and $17{,}608\,\textrm{s}$ (Velvet). See the Supplementary materials for results of all tested methods

$\mathsf{Method}$	$\mathsf{dist}_{\mathsf{MESSGMq}\alpha }$	$\mathsf {co-phylog}$	$\mathsf{Mash}$	$D_{2}$	$D_{2}^{*}$	$D_{2}^{q}$	$D_{2}^{q*}$	$\mathsf{dist}_{q}$ Velvet	$\mathsf{Time}$ ($\textrm{s},\ \mathsf{one thread}$)	$\mathsf{Time}\ (\textrm{s},\ \mathsf{parallel})$
$\mathsf{dist}_{\mathsf{MESSGMq}\alpha }$	1.000	0.831	0.943	0.683	0.867	0.684	0.868	1.000	8,908	NaN
$\mathsf {co-phylog}$	0.831	1.000	0.925	0.711	0.813	0.712	0.813	0.829	NaN	598
$\mathsf{Mash}$	0.943	0.925	1.000	0.734	0.877	0.735	0.878	0.942	NaN	500
$D_{2}$	0.683	0.711	0.734	1.000	0.949	1.000	0.949	0.683	3,289	NaN
$D_{2}^{*}$	0.867	0.813	0.877	0.949	1.000	0.950	1.000	0.866	3,329	NaN
$D_{2}^{q}$	0.684	0.712	0.735	1.000	0.950	1.000	0.949	0.684	3,302	NaN
$D_{2}^{q*}$	0.868	0.813	0.878	0.949	1.000	0.949	1.000	0.868	3,329	NaN
$\mathsf{dist}_{q}$ Velvet	1.000	0.829	0.942	0.683	0.866	0.684	0.868	1.000	88,156	4,276

### Results averaging

**Fig. 5:**
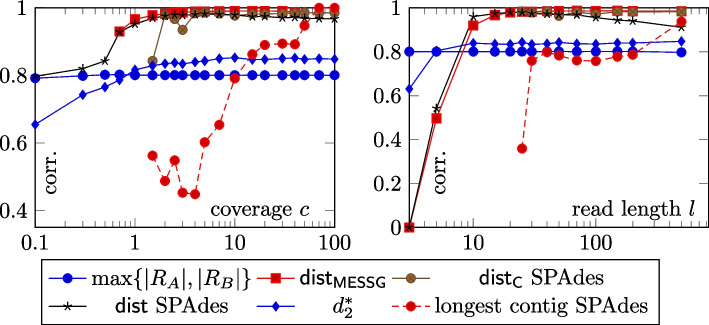
Plot of the average Pearson’s correlation coefficient for several choices of coverage (top plot) and read length (bottom plot) on the influenza dataset. See the Supplementary materials for the results on the various dataset

The influenza and various datasets contain reads for many coverages and read length values. This high range of coverage and read length was selected to show the behavior of the methods in extreme cases, for example in Fig. 5. For each coverage value (read length, respectively), we averaged the results. As extreme read length and coverage values are undesirable and likely to cause outliers, whenever we present an average, the average is calculated excluding the three most outlying values of read length (coverage). In such a case, the coverage spanned between 0.7 and 40, and the read length was between 15 and 100.

For both distance calculation time and Pearson’s correlation coefficient, we provide the average rank of the methods. This is because the time and correlation are hard to interpret, together with information on whether each algorithm calculated valid results within the time limit. Therefore, we sorted the results for each choice of coverage and read length, placing the methods that did not finish last together with the case when the correlation was not defined (i.e., all sequences were equidistant). Then the rank is defined as the number of better methods in the sorted list plus one. The rank was then averaged over coverage and read length values, as explained earlier.

## Discussion

**Fig. 6:**
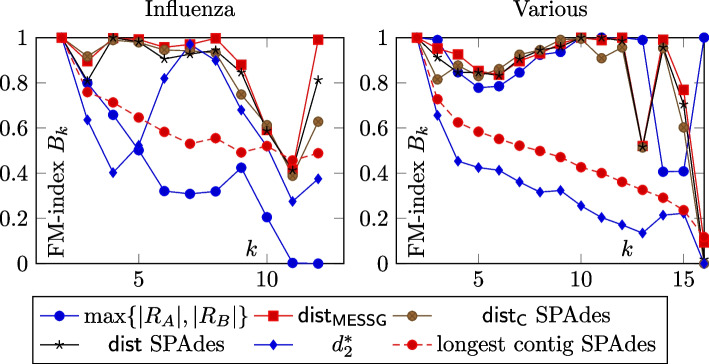
Plot of dependence of quality of the phylogenetic tree on depth in the tree. To calculate the Fowlkes-Mallows index $B_k$, the tree is cut at level *k* and the clusterings are compared

The main experimental results can be seen in Table 2. Besides that, Fig. 5 shows the dependency of the correlation between the reference and the predicted distances on read length and coverage. Table 3 shows experimental results on the e-coli dataset. Fig. 6 shows how the Fowlkes-Mallows index depends on the depth in the phylogenetic tree.

The results shown in Table 2 indicate that method $\mathsf{dist}_q$ including the efficiency optimizations from Efficiency improvements section, yields valid results in a broader range of cases than other methods. Compared to the alignment-free approaches, the method works well on both similar as well as dissimilar genomes, while the performance of the alignment-free approaches is worse on dissimilar genomes. Fig. 5 further illustrates that the method is capable of producing reasonable results for low-coverage data and very short reads.

Table 2 shows that compared to the previous versions of the method, the newly proposed changes are faster. However, the method is, as expected, slower than the alignment-free methods. Compared to the assembly time, there were differences in the order of magnitude. However, it needs to be said that the assembly algorithms were used with default parameters, which allowed parallel computation by default. If we look at the E.coli dataset in Table 3, we see that the single-threaded implementation is comparable to the assembly time and by the order of magnitude faster than assembly in the parallel version. Table 3 also shows that on 32 cores system, the speedup reached is more than 20 times.

Concerning the correlation, the method performs better on one dataset and worse on one dataset than our previous research. Compared to the alignment-free methods, the proposed method did better on two datasets while worse on one dataset.

The proposed changes improve the runtime up to three magnitudes. Note that in the case of the hepatitis dataset, the exact variant of the contig-based method finishes only in two cases, while the faster variant finishes in all cases.

The real-world experiments show that our method is successful in approximating the Levenshtein distance between the compared sequences. The experiments show that the method requires coverage of 2. Potential benefits, therefore, include a possible reduction of the wet lab sequencing, no need to use high coverages or to sequence mate pairs. The method is applicable to viral genomes or shorter bacteria genomes. Figure 5 has also shown that the correlation is high for read length of 10. This might be useful in some applications; for example, publication [17] justifies the need to use assembly-free, alignment-free, and reference-free tools by MiSeq [24] sequencing of rapidly mutating RNA viruses. Nevertheless, Fig. 5 shows that the possible conditions when our method works are much wider.

Our methods had two assumptions. The first one was that the read length *l* is the same for all reads. This can be justified by the fact that many of the sequencing technologies (including Illumina) read in each iteration a single nucleotide. As a result, all reads are sequenced with the same read length. The assumption that the coverage is equal for all samples is usually not met. Hence, in our publicly available implementation, this requirement is not enforced and can be replaced by either providing per-read-bag coverage or estimating the lengths of each genome. For our analyses, especially in the artificial datasets, we assumed that the reads are generated uniformly, which is usually not exactly true [36].

## Conclusion

We have presented a method capable of calculating sequence distances for inducing phylogenetic trees from reads and/or contigs of the input sequences. The method works universally for a wide range of coverage, read length, and similar and dissimilar organisms. Compared to the alignment-free approaches, the method turned out slower but performed better in terms of the correlation of the computed distance matrix with the ground truth. Also, the new method has a more straightforward interpretation in terms of the number of mutations needed to transform one genome into another.

## Supplementary Information

**Additional file 1.**

## Data Availability

Source codes and a pre-compiled version of the method are available on https://github.com/petrrysavy/reference-free-phylogeny.

## Footnotes

1 We adopt the assumption of contigs not overlapping in the original sequence from [31].

2 The resulting contigs and leftover reads may be concatenated in arbitrary order to produce the requested single superstring, which—due to the heuristic nature of the algorithm—is not necessarily the shortest.

3 Under the *molecular clock hypothesis*, this function indicates the least possible number of mutations, which, in turn, approximates the evolutionary distance.

4 The *q*-gram and *k*-mer represent the same thing — a substring of length *q* (*k*).

5 This number approximately matches free gap parameter *t* (which will be introduced in Read-read mapping section) for most common read length $l=100$ and coverage between 2 and 3.

6 Note that this function has the range [0, 1] while the unknown distance between *A* and *B* is in the range $[0, \max \{|A|, |B|\}]$; we will address the scale in a later step in Eq. (17).

7 See [28] for the derivation of the value assigned to *t*.
