# Supplementary material for: Reference-free phylogeny from sequencing data
Source: BioData Min. 2023 Mar 27;16:13. doi: 10.1186/s13040-023-00329-x (PMC10045052; doi:10.1186/s13040-023-00329-x)
Supplement: Supplementary file 1 — Additional file 1. [file 13040_2023_329_MOESM1_ESM.pdf]

Data	method	finished	assem. ms	distances ms	*rank* *dists.*	corr.	*rank* *corr.*	NJ B_4	NJ B_8	trip.d. NJ	UPGMA B_4	UPGMA B_8	trip.d. UPGMA
Influenza	reference	112/112	0	2,602	29.2	1	1	1	1	0	1	1	0
	$\max(\lvert R_A\rvert, \lvert R_B\rvert)$	112/112	0	335	**13.3**	.801	46.5	.658	.319	57	.67	.319	81
	$\mathsf{dist}_{\mathsf{MESSG}}(R_A, R_B)$	107/112	0	899,270	60.1	**.983**	**9.7**	.998	.997	5	1	1	23
	$\mathsf{dist}_{\mathsf{MESSGq}}(R_A, R_B)$	112/112	0	50,808	42.5	.966	27.9	.998	.967	28	.999	.977	33
	$\mathsf{dist}_{\mathsf{C}}(C_A, C_B)$ ABySS	87/112	21,628	17,469	44.7	.951	37.7	.983	.799	23	.983	.868	33
	$\mathsf{dist}_{\mathsf{C}}(C_A, C_B)$ Edena	72/112	285	18,483	47.2	.96	41.7	.996	.845	27	.997	.878	34
	$\mathsf{dist}_{\mathsf{C}}(C_A, C_B)$ SPAdes	43/112	13,529	22,661	56.8	.973	49.4	.989	.932	8	.994	.948	38
	$\mathsf{dist}_{\mathsf{C}}(C_A, C_B)$ SSAKE	68/112	2,079	17,735	48.5	.944	44.5	.974	.838	22	.972	.867	35
	$\mathsf{dist}_{\mathsf{C}}(C_A, C_B)$ Velvet	110/112	385	23,567	43.8	.958	31.6	.991	.906	28	.994	.91	5
	$\mathsf{dist}(T_A, T_B)$ ABySS	112/112	20,900	508,511	54.5	.979	17	.998	.997	14	.997	.998	19
	$\mathsf{dist}(T_A, T_B)$ Edena	112/112	233	430,385	53.4	.98	**15.1**	.998	.997	5	1	.997	19
	$\mathsf{dist}(T_A, T_B)$ SPAdes	112/112	12,380	625,883	56.7	**.983**	**8.9**	.998	.997	0	.997	1	19
	$\mathsf{dist}(T_A, T_B)$ SSAKE	112/112	1,655	552,860	53.4	**.98**	15.7	.998	.997	9	.997	.997	28
	$\mathsf{dist}(T_A, T_B)$ Velvet	111/112	378	749,033	57.9	.971	29.1	.998	.987	23	1	.994	5
	$\mathsf{dist}_q(T_A, T_B)$ ABySS	112/112	5,583	23,565	35.3	.963	32	1	.925	19	1	.934	38
	$\mathsf{dist}_q(T_A, T_B)$ Edena	112/112	264	16,090	35.6	.966	31.1	1	.942	28	1	.949	33
	$\mathsf{dist}_q(T_A, T_B)$ SPAdes	112/112	14,345	28,690	37.6	.971	23.1	1	.944	28	1	.954	23
	$\mathsf{dist}_q(T_A, T_B)$ SSAKE	112/112	2,302	27,515	36.3	.967	28.7	1	.951	19	1	.956	24
	$\mathsf{dist}_q(T_A, T_B)$ Velvet	112/112	446	22,478	37.7	.956	35.3	.998	.973	38	.996	.977	29
	Mash	112/112	0	**101**	**9**	.679	46.8	.438	.61	152	.476	.575	184
	d_2	112/112	0	335	17	.708	48.1	.427	.988	120	.303	.665	126
	d_2^*	112/112	0	389	18.3	.837	44.7	.402	.899	118	.378	.712	101
	d_2^{q*}	112/112	0	328	16.6	.631	50.3	.32	.272	160	.365	.105	196
	D_2	112/112	0	374	17.2	.443	59.2	.324	.001	171	.675	.316	118
	D_2^*	112/112	0	318	16.6	−.102	62.6	.436	.002	150	.504	.004	199
	D_2^{q*}	112/112	0	312	16.5	0	63.6	.32	.272	160	.365	.123	196
	d_2^q	112/112	0	**281**	15.2	.631	50.3	.32	.272	160	.365	.105	196
	D_2^q	112/112	0	327	16.5	0	63.6	.32	.272	160	.365	.123	196
	longest contig ABySS	87/112	21,628	1,182	26.4	.67	45.5	.621	.432	116	.629	.427	144
	longest contig Edena	72/112	285	1,055	33.3	.675	49.2	.624	.465	123	.636	.472	151
	longest contig SPAdes	43/112	13,529	1,465	48.2	.751	51.5	.713	.555	106	.743	.558	116
	longest contig SSAKE	68/112	2,079	785	32.5	.664	51	.606	.357	128	.594	.352	158
	longest contig Velvet	110/112	385	**38**	**7.5**	.569	53.8	.457	.23	133	.452	.234	167

Table 1: Average runtime, Pearson's correlation coefficient between the distance matrices, the Fowlkes-Mallows index for $k = 4$ and $k = 8$, and the triplets-distance between the trees on the *influenza* dataset. Note that the triplets distance is calculated only on a sample of read length and coverage values. The 'reference' method calculates the distances of the original sequences. For an explanation of the rank column, see the Experiments section of the paper. Note that the table is truncated for space reasons. Therefore, the rank columns show higher numbers than expected. The excluded rows mostly show the behavior of the presented methods on error-free idealized artificial contigs.

Data	method	finished	assem. ms	distances ms	*rank* *dists.*	corr.	*rank* *corr.*	NJ B_4	NJ B_8	trip.d. NJ	UPGMA B_4	UPGMA B_8	trip.d. UPGMA
Various	reference	112/112	0	57,099	16.9	1	1	1	1	0	1	1	0
	$\max(\lvert R_A\rvert, \lvert R_B\rvert)$	112/112	0	847	**4.1**	.907	14.1	.846	.924	48	.671	.655	352
	$\mathsf{dist}_{\mathsf{MESSG}}(R_A, R_B)$	64/112	0	1,299,980	24.8	**.933**	13	.925	.93	19	.622	.882	285
	$\mathsf{dist}_{\mathsf{MESSGq}}(R_A, R_B)$	109/112	0	605,647	20	.927	**8.7**	.838	.973	42	.659	.768	316
	$\mathsf{dist}_{\mathsf{C}}(C_A, C_B)$ ABySS	72/112	7,959	779,370	22.4	.921	14.7	.843	.926	54	.629	.753	306
	$\mathsf{dist}_{\mathsf{C}}(C_A, C_B)$ Edena	57/112	679	1,070,977	23.8	.92	16.9	.858	.932	39	.629	.773	310
	$\mathsf{dist}_{\mathsf{C}}(C_A, C_B)$ SPAdes	58/112	7,342	899,891	22.9	.923	15.5	.857	.944	40	.635	.798	294
	$\mathsf{dist}_{\mathsf{C}}(C_A, C_B)$ SSAKE	108/112	1,235	749,197	20.7	.928	**5.4**	.839	.922	25	.632	.902	241
	$\mathsf{dist}_{\mathsf{C}}(C_A, C_B)$ Velvet	34/112	17,783	1,239,632	25.5	.917	19.8	.877	.945	16	.638	.838	292
	$\mathsf{dist}(T_A, T_B)$ ABySS	70/112	5,086	1,684,468	24.7	.928	13.2	.862	.923	27	.626	.82	292
	$\mathsf{dist}(T_A, T_B)$ Edena	69/112	168	1,681,308	24.6	**.932**	12.3	.916	.934	18	.629	.876	285
	$\mathsf{dist}(T_A, T_B)$ SPAdes	70/112	3,449	1,666,859	24.1	.932	12.2	.913	.931	20	.63	.874	277
	$\mathsf{dist}(T_A, T_B)$ SSAKE	64/112	568	1,635,059	26.1	.919	12.9	.831	.909	27	.611	.892	242
	$\mathsf{dist}(T_A, T_B)$ Velvet	67/112	13,897	1,584,465	24.9	.932	12.4	.92	.929	19	.623	.882	286
	$\mathsf{dist}_q(T_A, T_B)$ ABySS	110/112	10,937	252,197	16.5	.919	11.7	.85	.932	39	.65	.755	322
	$\mathsf{dist}_q(T_A, T_B)$ Edena	112/112	790	360,304	15.9	.921	10.9	.843	.941	44	.661	.752	321
	$\mathsf{dist}_q(T_A, T_B)$ SPAdes	110/112	6,197	316,445	16.2	.922	10.7	.852	.941	49	.65	.766	320
	$\mathsf{dist}_q(T_A, T_B)$ SSAKE	111/112	2,231	428,540	17.9	**.934**	**6.4**	.844	.954	62	.726	.847	227
	$\mathsf{dist}_q(T_A, T_B)$ Velvet	110/112	19,583	355,127	16.3	.922	10.1	.845	.945	58	.646	.765	318
	Mash	84/112	0	**562**	8.3	.664	17.8	.464	.342	344	.396	.315	394
	d_2	109/112	0	741	8.7	.269	23.8	.469	.355	332	.358	.41	364
	d_2^*	110/112	0	756	8.5	.442	20.1	.453	.316	308	.378	.19	368
	d_2^{q*}	109/112	0	**721**	8	.573	17.4	.32	.28	399	.446	.099	462
	D_2	107/112	0	739	8.4	.294	22.5	.463	.067	404	.671	.641	360
	D_2^*	110/112	0	734	7.8	.291	22.6	.474	.114	386	.604	.428	436
	D_2^{q*}	110/112	0	774	7.7	0	25	.32	.28	399	.446	.122	462
	d_2^q	109/112	0	760	7.6	.573	17.4	.32	.28	399	.446	.099	462
	D_2^q	111/112	0	743	**7.4**	0	25	.32	.28	399	.446	.122	462
	longest contig ABySS	72/112	7,959	6,439	13.6	.562	20.1	.495	.345	298	.512	.443	429
	longest contig Edena	57/112	679	18,206	17.8	.571	20.8	.537	.389	333	.542	.452	445
	longest contig SPAdes	58/112	7,342	14,861	15.6	.626	20.1	.533	.377	323	.548	.465	420
	longest contig SSAKE	108/112	1,235	**385**	**3.5**	.386	20.9	.482	.433	349	.448	.166	368
	longest contig Velvet	34/112	17,783	34,858	22.5	.681	21.4	.625	.498	329	.632	.614	440

Table 2: Average runtime, Pearson's correlation coefficient between the distance matrices, the Fowlkes-Mallows index for $k = 4$ and $k = 8$, and the triplets-distance between the trees on the *various* dataset. Note that the triplets distance is calculated only on a sample of read length and coverage values. The table was generated under the same conditions as Table 1.

Data	method	finished	assem. (ms)	distances (ms)	*rank* / *dists.*	corr.	*rank* / *corr.*	NJ / B_4	NJ / B_8	trip.d. / NJ	UPGMA / B_4	UPGMA / B_8	trip.d. / UPGMA
Hepatitis	reference	9/9	0	1,748,984	16.9	1	1	1	1	0	1	1	0
	$\max(\lvert R_A\rvert, \lvert R_B\rvert)$	9/9	0	29,340	5.8	.181	19.3	.724	.828	24,017	.553	.368	48,325
	$\mathsf{dist}_{\mathsf{MESSG}}(R_A, R_B)$	9/9	0	42,332,682	21.1	.965	8.3	1	.904	4,407	.99	.954	3,076
	$\mathsf{dist}_{\mathsf{MESSGq}\alpha}(R_A, R_B)$	9/9	0	1,118,585	15.4	.897	14.2	1	.935	4,543	.913	.942	6,974
	$\mathsf{dist}_{\mathsf{C}}(C_A, C_B)$ ABySS	9/9	35,145	48,256,963	22.3	.949	9.9	1	.912	13,321	.948	.937	5,344
	$\mathsf{dist}_{\mathsf{C}}(C_A, C_B)$ Edena	9/9	7,038	44,548,818	21.2	.892	**5.1**	1	.839	10,035	.954	.912	4,625
	$\mathsf{dist}_{\mathsf{C}}(C_A, C_B)$ SPAdes	2/9	76,514	31,517,537	23.1	.869	20.6	1	.893	7,361	.87	.931	379
	$\mathsf{dist}_{\mathsf{C}}(C_A, C_B)$ SSAKE	5/9	69,156	55,880,178	23.2	.901	15.1	1	.945	11,055	.976	.947	7,955
	$\mathsf{dist}_{\mathsf{C}}(C_A, C_B)$ Velvet	4/9	11,090	59,898,794	23.9	**.98**	14.6	1	.988	2,419	1	.974	2,128
	$\mathsf{dist}(T_A, T_B)$ ABySS	0/9	NaN	NaN	24.4	NaN	24.4	NaN	NaN	NaN	NaN	NaN	NaN
	$\mathsf{dist}(T_A, T_B)$ Edena	0/9	NaN	NaN	24.4	NaN	24.4	NaN	NaN	NaN	NaN	NaN	NaN
	$\mathsf{dist}(T_A, T_B)$ SPAdes	0/9	NaN	NaN	24.4	NaN	24.4	NaN	NaN	NaN	NaN	NaN	NaN
	$\mathsf{dist}(T_A, T_B)$ SSAKE	0/9	NaN	NaN	24.4	NaN	24.4	NaN	NaN	NaN	NaN	NaN	NaN
	$\mathsf{dist}(T_A, T_B)$ Velvet	0/9	NaN	NaN	24.4	NaN	24.4	NaN	NaN	NaN	NaN	NaN	NaN
	$\mathsf{dist}_{q\alpha}(T_A, T_B)$ ABySS	9/9	48,194	520,227	12.7	.957	10.6	1	.932	13,051	.823	.89	12,004
	$\mathsf{dist}_{q\alpha}(T_A, T_B)$ Edena	9/9	12,889	520,091	12.8	.929	10.3	.976	.942	11,057	.835	.823	14,027
	$\mathsf{dist}_{q\alpha}(T_A, T_B)$ SPAdes	9/9	130,268	373,244	11.7	.911	13.6	.966	.843	9,366	.75	.862	16,600
	$\mathsf{dist}_{q\alpha}(T_A, T_B)$ SSAKE	9/9	88,516	615,615	14.2	.901	12.9	.961	.937	13,710	.851	.862	16,074
	$\mathsf{dist}_{q\alpha}(T_A, T_B)$ Velvet	9/9	27,814	1,729,999	16.9	.955	7.9	1	.994	5,939	.934	.941	7,126
	Mash	9/9	0	**2,350**	**1.4**	.967	8.1	1	.918	9,532	.964	.966	5,351
	d_2	7/9	0	**27,145**	9.1	**.973**	10.1	1	.864	5,370	.982	.963	8,532
	d_2^*	7/9	0	28,189	10.9	.972	10.4	1	.902	8,113	.893	.972	9,374
	d_2^{q*}	9/9	0	29,296	**6.4**	.972	**6.8**	1	.896	8,339	.894	.973	9,646
	D_2	8/9	0	30,458	8.2	$-.181$	21.2	.93	.429	57,198	.662	.545	61,061
	D_2^*	7/9	0	27,718	9.2	$-.783$	22.9	.929	.407	57,268	.662	.545	61,611
	D_2^{q*}	9/9	0	27,151	**4.6**	$-.782$	23.4	.922	.404	57,384	.662	.545	61,544
	d_2^q	9/9	0	27,885	6.7	**.973**	**5.1**	1	.87	5,347	.984	.96	8,613
	D_2^q	7/9	0	31,481	12.1	$-.187$	22.3	.931	.415	57,499	.662	.545	61,085
	longest contig ABySS	9/9	35,145	2,493,455	18.2	.53	18.1	.946	.685	32,211	.654	.686	37,805
	longest contig Edena	9/9	7,038	1,581,613	15.6	.515	17.8	.918	.76	23,452	.783	.8	27,115
	longest contig SPAdes	2/9	76,514	3,242,365	21.1	.395	23	.867	.776	1,549	.822	.775	7,460
	longest contig SSAKE	5/9	69,156	764,321	16.7	.334	21	.862	.624	22,595	.712	.661	30,597
	longest contig Velvet	4/9	11,090	**515**	13.1	.296	21.3	.919	.473	51,443	.637	.58	55,159

Table 3: Average runtime, Pearson's correlation coefficient between the distance matrices, the Fowlkes-Mallows index for $k = 4$ and $k = 8$, and the triplets-distance between the trees on the *hepatitis* dataset. The table was generated under the same conditions as Table 1.

Data	method	finished	assem. (ms)	distances (ms)	*rank* / *dists.*	corr.	*rank* / *corr.*	NJ / B_4	NJ / B_8	trip.d. / NJ	UPGMA / B_4	UPGMA / B_8	trip.d. / UPGMA
Chroms	reference	1/1	0	668,767	20	1	1	1	1	0	1	1	0
	$\max(\lvert R_A\rvert, \lvert R_B\rvert)$	1/1	0	2,184	13	.331	18	.613	.298	880	.64	.404	1,019
	$\mathsf{dist}_{\mathsf{MESSG}}(R_A, R_B)$	1/1	0	23,758,416	24	.848	14	.585	.26	923	.408	.227	1,004
	$\mathsf{dist}_{\mathsf{MESSGq}\alpha}(R_A, R_B)$	1/1	0	202,517	19	.825	15	.9	.247	939	.404	.227	1,069
	$\mathsf{dist}_{\mathsf{C}}(C_A, C_B)$ ABySS	0/1	NaN	NaN	27	NaN	25	NaN	NaN	-1	NaN	NaN	-1
	$\mathsf{dist}_{\mathsf{C}}(C_A, C_B)$ Edena	0/1	NaN	NaN	27	NaN	25	NaN	NaN	-1	NaN	NaN	-1
	$\mathsf{dist}_{\mathsf{C}}(C_A, C_B)$ SPAdes	0/1	NaN	NaN	27	NaN	25	NaN	NaN	-1	NaN	NaN	-1
	$\mathsf{dist}_{\mathsf{C}}(C_A, C_B)$ SSAKE	0/1	NaN	NaN	27	NaN	25	NaN	NaN	-1	NaN	NaN	-1
	$\mathsf{dist}_{\mathsf{C}}(C_A, C_B)$ Velvet	0/1	NaN	NaN	27	NaN	25	NaN	NaN	-1	NaN	NaN	-1
	$\mathsf{dist}(T_A, T_B)$ ABySS	1/1	17,838	24,085,638	25	.911	6	.638	.342	707	.774	.307	752
	$\mathsf{dist}(T_A, T_B)$ Edena	1/1	1,063	23,404,639	22	.879	12	.676	.211	968	.553	.334	1,002
	$\mathsf{dist}(T_A, T_B)$ SPAdes	1/1	22,898	23,757,934	23	.873	13	.676	.211	968	.553	.334	1,002
	$\mathsf{dist}(T_A, T_B)$ SSAKE	1/1	51,604	20,576,658	21	.903	7	.805	.342	948	.359	.334	1,053
	$\mathsf{dist}(T_A, T_B)$ Velvet	1/1	7,866	24,668,207	26	.902	8	.585	.26	923	.619	.334	1,002
	$\mathsf{dist}_{q\alpha}(T_A, T_B)$ ABySS	1/1	17,838	144,725	18	.914	5	.805	.211	989	.488	.334	807
	$\mathsf{dist}_{q\alpha}(T_A, T_B)$ Edena	1/1	1,063	126,282	14	.887	9	.805	.211	987	.455	.334	897
	$\mathsf{dist}_{q\alpha}(T_A, T_B)$ SPAdes	1/1	22,898	127,061	16	.881	11	.805	.329	991	.553	.208	1,008
	$\mathsf{dist}_{q\alpha}(T_A, T_B)$ SSAKE	1/1	51,604	126,565	15	**.914**	**4**	.805	.211	987	.819	.43	597
	$\mathsf{dist}_{q\alpha}(T_A, T_B)$ Velvet	1/1	7,866	127,200	17	.881	10	.805	.222	991	.553	.208	1,002
	Mash	1/1	0	**173**	**3**	.33	19	.599	.382	787	.588	.307	1,060
	d_2	1/1	0	696	5	.258	22	.706	.368	756	.503	.497	877
	d_2^*	1/1	0	697	6	.301	20	.805	.303	928	.503	.328	1,050
	d_2^{q*}	1/1	0	697	6	**.959**	**2**	.805	.316	1,083	.519	.283	1,120
	D_2	1/1	0	692	4	$3.933 \cdot 10^{-2}$	23	.9	.283	963	.64	.404	1,026
	D_2^*	1/1	0	1,018	11	$2.919 \cdot 10^{-2}$	24	.852	.273	967	.471	.358	1,158
	D_2^{q*}	1/1	0	714	8	0	25	.805	.316	108	.519	.283	1,120
	d_2^q	1/1	0	734	9	**.959**	**2**	.805	.316	1,083	.519	.283	1,120
	D_2^q	1/1	0	1,010	10	0	25	.805	.316	108	.519	.283	1,120
	longest contig ABySS	1/1	17,838	1,213	12	.34	17	.706	.364	1,016	.55	.38	1,089
	longest contig Edena	0/1	NaN	NaN	27	NaN	25	NaN	NaN	-1	NaN	NaN	-1
	longest contig SPAdes	0/1	NaN	NaN	27	NaN	25	NaN	NaN	-1	NaN	NaN	-1
	longest contig SSAKE	1/1	51,604	**152**	**2**	.297	21	.588	.302	964	.66	.538	977
	longest contig Velvet	1/1	7,866	**31**	**1**	.574	16	.805	.404	1,007	.519	.158	1,307

Table 4: Average runtime, Pearson's correlation coefficient between the distance matrices, the Fowlkes-Mallows index for $k = 4$ and $k = 8$, and the triplets-distance between the trees on the *chroms* dataset. The table was generated under the same conditions as Table 1.

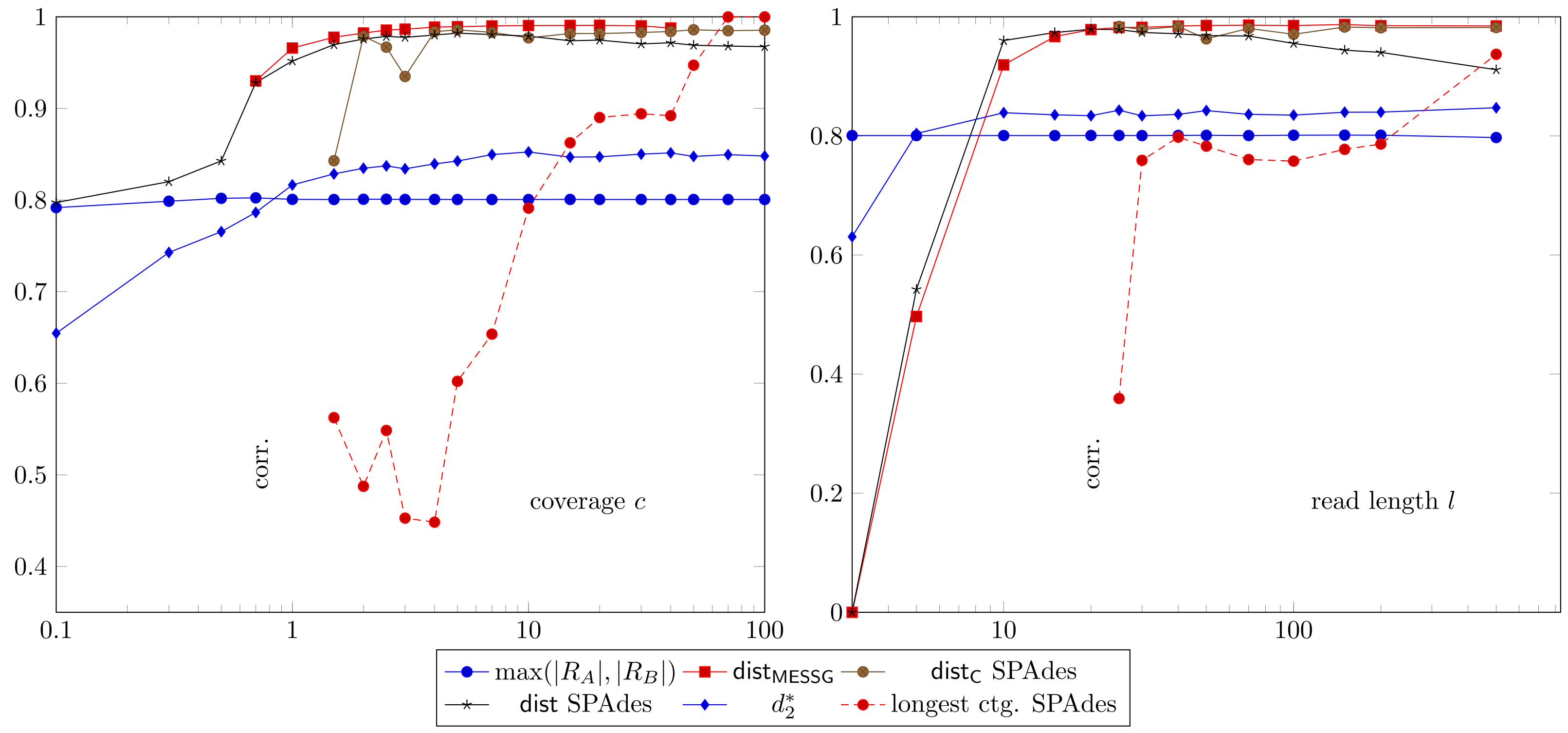

Figure 1: Plot of average Pearson's correlation coefficient for several choices of coverage (top plot) and read length (bottom plot) on the influenza dataset. See the supplementary materials for the results on the various dataset.

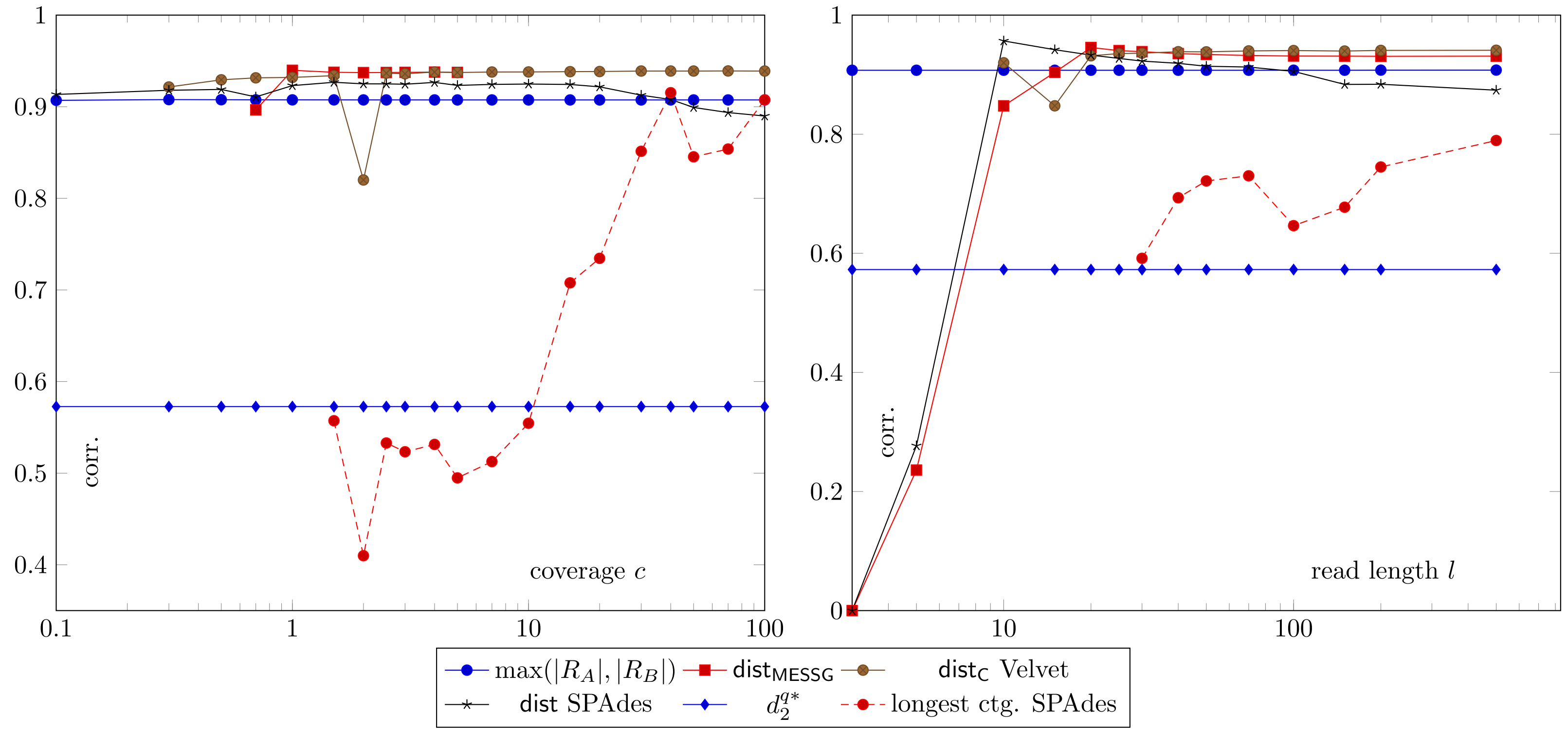

Figure 2: Plot of average Pearson's correlation coefficient for several choices of coverage (top plot) and read length (bottom plot) on the various dataset.

Table 5: Pairwise correlations of distance matrices on "E. coli" dataset.

	$\mathsf{dist}_{\mathsf{MESSGq}\alpha}$	$\mathsf{dist}_{\mathsf{MESSGMq}\alpha}$	co-phylog	Mash	d_2	d_2^*	d_2^q	d_2^{q*}	D_2	D_2^*	D_2^q	D_2^{q*}	dist_q ABySS	dist_q Edena	dist_q SSAKE	dist_q Velvet
$\mathsf{dist}_{\mathsf{MESSGq}\alpha}$	1.000	1.000	0.831	0.943	0.269	0.302	0.269	0.304	0.683	0.867	0.684	0.868	1.000	1.000	1.000	1.000
$\mathsf{dist}_{\mathsf{MESSGMq}\alpha}$	1.000	1.000	0.831	0.943	0.269	0.302	0.269	0.304	0.683	0.867	0.684	0.868	1.000	1.000	1.000	1.000
co-phylog	0.831	0.831	1.000	0.925	0.161	0.215	0.161	0.216	0.711	0.813	0.712	0.813	0.829	0.829	0.829	0.829
Mash	0.943	0.943	0.925	1.000	0.286	0.339	0.286	0.341	0.734	0.877	0.735	0.878	0.942	0.942	0.942	0.942
d_2	0.269	0.269	0.161	0.286	1.000	0.956	1.000	0.956	0.240	0.246	0.243	0.249	0.270	0.270	0.271	0.270
d_2^*	0.302	0.302	0.215	0.339	0.956	1.000	0.956	1.000	0.322	0.303	0.325	0.307	0.302	0.302	0.303	0.302
d_2^q	0.269	0.269	0.161	0.286	1.000	0.956	1.000	0.956	0.240	0.246	0.243	0.249	0.270	0.270	0.271	0.270
d_2^{q*}	0.304	0.304	0.216	0.341	0.956	1.000	0.956	1.000	0.323	0.305	0.326	0.308	0.304	0.304	0.305	0.304
D_2	0.683	0.683	0.711	0.734	0.240	0.322	0.240	0.323	1.000	0.949	1.000	0.949	0.683	0.683	0.683	0.683
D_2^*	0.867	0.867	0.813	0.877	0.246	0.303	0.246	0.305	0.949	1.000	0.950	1.000	0.866	0.866	0.866	0.866
D_2^q	0.684	0.684	0.712	0.735	0.243	0.325	0.243	0.326	1.000	0.950	1.000	0.949	0.684	0.684	0.684	0.684
D_2^{q*}	0.868	0.868	0.813	0.878	0.249	0.307	0.249	0.308	0.949	1.000	0.949	1.000	0.868	0.868	0.868	0.868
dist_q ABySS	1.000	1.000	0.829	0.942	0.270	0.302	0.270	0.304	0.683	0.866	0.684	0.868	1.000	1.000	1.000	1.000
dist_q Edena	1.000	1.000	0.829	0.942	0.270	0.302	0.270	0.304	0.683	0.866	0.684	0.868	1.000	1.000	1.000	1.000
dist_q SSAKE	1.000	1.000	0.829	0.942	0.271	0.303	0.271	0.305	0.683	0.866	0.684	0.868	1.000	1.000	1.000	1.000
dist_q Velvet	1.000	1.000	0.829	0.942	0.270	0.302	0.270	0.304	0.683	0.866	0.684	0.868	1.000	1.000	1.000	1.000

Table 6: Runtime on "E. coli" dataset. Assembly time (without distance matrix calculation) on the same dataset is 24,980 s (ABySS), 17,514 s (Edena), 1021,184 s (SPAdes), 229,350 s (SSAKE), and 17,608 s (Velvet).

Method	Time (s, one thread)	Time (s, parallel)
$\mathsf{dist}_{\mathsf{MESSGq}\alpha}$	8,908	NaN
$\mathsf{dist}_{\mathsf{MESSGMq}\alpha}$	8,908	NaN
co-phylog	NaN	598
Mash	NaN	500
d_2	3,343	NaN
d_2^*	3,311	NaN
d_2^q	3,331	NaN
d_2^{q*}	3,346	NaN
D_2	3,289	NaN
D_2^*	3,329	NaN
D_2^q	3,302	NaN
D_2^{q*}	3,329	NaN
dist_q ABySS	75,427	4,305
dist_q Edena	68,326	4,382
dist_q SPAdes	NaN	NaN
dist_q SSAKE	82,604	4,575
dist_q Velvet	88,156	4,276
